# Impact damage to the middle trough of a scraper conveyor based on the engineering discrete element method and orthogonal matrix analysis

**DOI:** 10.1371/journal.pone.0266831

**Published:** 2022-04-18

**Authors:** Yanping Yao, Weili Liu, Zhipeng Gao

**Affiliations:** 1 Taiyuan University of Science and Technology, Taiyuan, Shanxi, China; 2 Taiyuan Institute of China Coal Technology & Engineering Corp, Taiyuan, Shanxi, China; 3 XCMG Construction Machinery Co., Ltd., Xuzhou, Jiangsu, China; Semnan University, ISLAMIC REPUBLIC OF IRAN

## Abstract

The middle trough serves as a key part of a scraper conveyor. During the working process, falling raw coal lands on the middle plate of the trough, causing impact damage. This study aims to find the optimal working condition combination to minimize impact damage to the middle trough based on the engineering discrete element method (EDEM) and orthogonal matrix analysis (OMA). In EDEM software, simulation data of the impact damage depth and normal cumulative contact energy of the middle trough corresponding to the four influencing factors of the transverse laying roll angle, front lean angle, raw coal particle size, and chain layout and spacing under different horizontal conditions are obtained. Matrices of the impact damage depth and normal cumulative contact energy are separately established. Based on the respective factor layer, level and evaluation index weight matrices, a global weight matrix is finally obtained. The optimal combination of working conditions is obtained, and the weight of each factor on impact damage to the middle trough is determined by the weight coefficient. The accuracy of the simulation results is then verified in experiments. Among the considered factors, the raw coal particle size achieves the highest impact damage coefficient. When the raw coal particle size is the smallest (0.5 times the basic particle size), the transverse roll angle and front lean angle of the middle trough are positive (5° and 10°, respectively), the chain adopts the double-center chain arrangement, and minimal impact damage to the middle trough occurs. OMA reduces the test times to determine the optimal working conditions of a scraper conveyor.

## Introduction

A scraper conveyor constitutes the core equipment in a fully mechanized mining system, which undertakes important tasks, such as shearer haulage direction, hydraulic support movement and support and coal transportation [1]. The body of the scraper conveyor is composed of several sections of the middle trough, which is welded along the trough side and middle plate. The middle trough is one of the key components of a scraper conveyor. During operation, raw coal cut by the shearer impacts the middle trough from different heights and angles. In this process, due to the impact of falling raw coal as well as the sliding friction between raw coal, scraper and scraper chains, the surface material of the middle plate can become detached, forming impact damage and wear. Ultimately, the middle trough may be damaged. According to the literature, no less than 80% of all operation faults of scraper conveyors are caused by middle trough damage [2–4]. Therefore, prolonging the service life of the middle trough and improving the fully mechanized mining efficiency have attracted extensive attention from scholars in related fields.

To improve the quality of scraper conveyors and prolong their service life, scholars have invested numerous efforts. For instance, in terms of impact, scholars have analyzed the characteristics of impact damage to conveyors under impact loads and provided a method for impact energy calculation [5, 6]. In terms of fatigue, scholars have investigated fatigue crack propagation, based on which a novel crack propagation theory has been established [7, 8]. In terms of simulation model construction, researchers have proposed a discrete element impact calculation program based on the sphere model, which provides a new impact research method [9]. In terms of discrete elements, relevant software has been employed to analyze the effects of different scraper chain speeds, different falling heights of coal particles, varying impact loads and varying scraper laying lean angles on the wear patterns of the middle trough as well as conveyor efficiency [10, 11]. Regarding design, scholars have conducted research on the effects of the scraper spacing, impact velocity of bulk materials and scraper chain speed on wear of the middle trough [12]. Considering composite factors, scholars have analyzed the characteristics of impact damage to the middle trough under the combined action of coal properties, coal impact height and conveying speed based on orthogonal tests, and the weight of each factor has been obtained [13]. The influence of the load, silicon carbide abrasive particle size, sliding speed and sliding distance on the roughness of the wear surface of cast 7075 aluminum alloy has been determined [14–16]. The influence of multiple evaluation indices, such as cutting medium mechanical parameters, roller rotatory speed and impact frequency, on the working performance of point impact cutting rollers has been analyzed with the numerical analysis method [17]. In addition, scholars have studied the wear characteristics of the middle trough of a scraper conveyor. Through Plackett–Burman experiments, it has been found that the interaction between the waste rock content, moisture content and normal load can aggravate wear of the middle trough, and a regression prediction model of the wear amount has been established [18, 19].

The operation conditions of scraper conveyors are complex, and the middle trough tends to suffer early damage under the impact of raw coal and wear of scraper chains due to raw coal. Wear of the scraper chains of the middle trough has been widely studied using finite element analysis. By changing the number of sprocket teeth, chain pitch and operation speed, this problem has been satisfactorily overcome [20, 21]. However, the middle trough is subjected to the combined action of multiple factors; i.e., this part is also affected by coal fall, scraper conveyor layout and chain spacing, in addition to friction and wear. Different combinations of these factors result in different scraper conveyor working conditions. To date, most existing studies have been single-factor studies, and there have been few studies considering the combined action of multiple factors. Although [13] is a multifactor study, it is based on the properties of raw coal. In a fully mechanized mining system, the properties of raw coal are uncontrollable factors. Therefore, the above study provides no notable significance for prolonging the service life of the middle trough. Therefore, to prolong the service life of the middle trough, the key point is to control the working conditions of the middle trough.

Based on the aforementioned issues, this study aims to simulate and analyze the four influencing factors of the middle trough, i.e., roll angle, front lean angle, raw coal particle size and chain spacing, to obtain the impact damage depth and normal cumulative contact energy of the middle trough under these different factors. Based on the simulation results, orthogonal matrix analysis (OMA) [22] is employed to obtain the optimal combination working conditions. The accuracy of the results is then verified through experiments. The results of this study may provide a theoretical basis for the design and layout of the middle trough of scraper conveyors.

## Methods

In actual practice, impact damage to the middle trough of a scraper conveyor is caused by the combined action of various factors. If a comprehensive test of multiple factors is carried out, the work burden will be tremendous, which will make the task very difficult to implement. However, the very large amount of work can be greatly reduced by using the orthogonal test method.

### Basic theories

#### OMA

An orthogonal table of the above four factors at three levels is constructed (Table 1).

**Table 1:** Orthogonal table of the four factors considering three levels.

Level	Factor			
	A	B	C	D
1	A_1_	B_1_	C_1_	D_1_
2	A_2_	B_2_	C_2_	D_2_
3	A_3_	B_3_	C_3_	D_3_

Simulation analysis based on four factors at three levels requires 3^4^ = 81 tests. Adoption of the orthogonal method allows some test data to replace the required, comprehensive tests. Therefore, simulation results can be efficiently obtained. The orthogonal test table is summarized in Table 2.

**Table 2:** Orthogonal test table.

Test no.	Factor			
	A	B	C	D
1	A_1_	B_1_	C_1_	D_1_
2	A_1_	B_2_	C_2_	D_2_
3	A_1_	B_3_	C_3_	D_3_
4	A_2_	B_1_	C_2_	D_3_
5	A_2_	B_2_	C_3_	D_1_
6	A_2_	B_3_	C_1_	D_2_
7	A_3_	B_1_	C_3_	D_2_
8	A_3_	B_2_	C_1_	D_3_
9	A_3_	B_3_	C_2_	D_1_

In an orthogonal test with *m* factors at *n* levels, the average value of the evaluation index of the *i* factor at the *j* level is ${\bar{P}}_{ij}$. Under the condition of $p_{ij}={\bar{P}}_{ij}$, an index matrix is constructed as follows [22]:

$$P=[\begin{array}{ccccc} p_{11} & 0 & 0 & \cdots & 0\\ p_{12} & 0 & 0 & \cdots & 0\\ \cdots & \cdots & \cdots & \cdots & \cdots \\ p_{1n} & 0 & 0 & \cdots & 0\\ 0 & p_{21} & 0 & \cdots & 0\\ 0 & p_{22} & 0 & \cdots & 0\\ \cdots & \cdots & \cdots & \cdots & \cdots \\ 0 & p_{2n} & 0 & \cdots & 0\\ \cdots & \cdots & \cdots & \cdots & \cdots \\ 0 & 0 & 0 & \cdots & p_{m1}\\ 0 & 0 & 0 & \cdots & p_{m2}\\ \cdots & \cdots & \cdots & \cdots & \cdots \\ 0 & 0 & 0 & \cdots & p_{mn} \end{array}]$$

We let $W_{i}=\frac{1}{\sum_{j=1}^{n}\frac{1}{p_{ij}}}$, and a factor-layer matrix is obtained as follows [22]:

$$W=[\begin{array}{cccc} W_{1} & 0 & \cdots & 0\\ 0 & W_{2} & \cdots & 0\\ \cdots & \cdots & \cdots & \cdots \\ 0 & 0 & 0 & W_{m} \end{array}]$$

Supposing that the range of factor *A*_*i*_ is *x*_*i*_ and that its weight among all factors is $X_{i}=\frac{x_{i}}{\sum_{i=1}^{m}x_{i}}$, a horizontal layer matrix is obtained as follows [22]:

$$X={\left[\begin{array}{cccc} X_{1} & X_{2} & \ldots & X_{m} \end{array}\right]}^{T}$$

Then, a weight matrix of the evaluation indices is constructed as follows [22]:

$$G=PWX={\left[\begin{array}{cccc} G_{1} & G_{2} & \cdots & G_{n} \end{array}\right]}^{T}$$

where $G_{1}=p_{11}W_{1}X_{1}^{}$ denotes the weight of factor A at the first level among all evaluation indices at all levels.

#### Data of the four factors at the three levels

A scraper conveyor completes sliding under the pushing force of hydraulic support. Due to unevenness of the ground, horizontal, leftward or rightward inclination of the middle trough in the transverse direction occurs, and actual working conditions, such as horizontal, uphill or downhill transportation in the vertical direction, occur. Additionally, the chains exhibit layouts of middle double chains, quasi-edge double chains, edge double chains, etc. In actual practice, impact damage to the middle slot of the scraper conveyor can also be caused by the waste rock content, apart from the transverse laying roll angle, front lean angle, raw coal particle size, and chain layout and spacing. However, due to the uncontrollable nature of the waste rock content, this factor is not considered in this study. Therefore, in this study, four factors are considered at three levels (Table 3), which include 1) the transverse laying roll angle: 0°, 5° and -5°; 2) front lean angle: 0°, 10° and -10°; 3) chain layout and spacing: middle double-chain layout with a spacing of 140 mm, quasi-edge double-chain layout with a spacing of 420 mm and edge double-chain layout with a spacing of 700 mm; and 4) coal particle size: 35 mm, 70 mm and 105 mm (0.5-, 1.0- and 1.5-fold, respectively, the basic coal particle size (70 mm)).

**Table 3:** Four factors at the three levels for impact damage.

Level	Factor			
	A	B	C	D
	Transverse roll angle (°)	Front lean angle (°)	Coal particle size (mm)	Chain spacing (mm)
1	0	0	35	140
2	-5	-10	70	420
3	5	10	105	700

### Simulation experiment

The middle trough is impacted by falling coal, and its surface material can fall off to form impact pits. In this study, the impact damage due to raw coal to the middle plate of the middle trough is characterized by the volume of impact pits.

#### Impact damage theory [21]

The volume of impact pits *△V* is calculated as follows:

$$\Delta V=\frac{\pi }{3}h^{2}(3R-h)+[\frac{\pi R^{2}}{180}\mathrm{arc}\;\sin\frac{d}{2R}-\frac{d(R-h)}{2}]l$$ (1)

where *d* denotes the contact diameter of the raw coal with the middle plate (mm), *L* denotes the scratch length of the impact damage (mm), *h* denotes the depth of the impact pit (mm) and *R* denotes the radius of the coal particle (mm).

In the process of raw coal impacting the middle plate, the impact load on the middle plate is decomposed into the normal impact force *F*_*n*_ and the tangential impact force *F*_*τ*_. The relationship between these forces is as follows:

$$F_{\tau }=\mu F_{n}$$ (2)

where *μ* denotes the friction coefficient between the raw coal and middle plate.

According to the conservation of energy, the following can be obtained:

$$-(F_{\tau }-mg)l=\frac{1}{2}mv_{\tau }^{2}(\kappa_{\tau }^{2}-1)$$ (3)

where *m* denotes the mass of the raw coal particles (kg), *v*_*τ*_ denotes the tangential velocity of the raw coal (m/s), and *κ*_*τ*_ denotes the tangential restitution coefficient of the impact force of the raw coal on the middle plate, among which the following applies:

$$v_{\tau }=v\cos\alpha ;\;\kappa_{\tau }=1-\mu (1+\kappa_{n})\tan\alpha ;$$

$$\kappa_{n}={\left\{\frac{6\sqrt{3}}{5}[1-\frac{1}{6}{\left(\frac{v_{y}}{v_{n}}\right)}^{2}]\right\}}^{1/2}\cdot {\left[\frac{v_{y}/v_{n}}{v_{y}/v_{n}+\sqrt{1.2-0.2{\left(v_{y}/v_{n}\right)}^{2}}}\right]}^{1/4}$$

where *a* is the angle between the incident direction and the horizontal plane when raw coal impacts the middle plate (°), *k*_*n*_ is the normal restitution coefficient of the impact of raw coal on the middle plate, and *v*_*y*_ is the normal minimum velocity when the middle plate yields (m/s).

We suppose that the yielding process of the contact surface of the middle plate satisfies the Mohr–Coulomb criteria:

$$v_{y}=18.1\frac{R^{3/2}}{E^{2}m_{2}^{1/2}}{\left(\frac{2k\cos\beta }{K_{1}-K_{2}\sin\beta }\right)}^{5/2}$$ (4)

where *k* is the cohesion force of the middle plate (N) and *E* is the equivalent elastic modulus (MPa), among which:

$$\frac{1}{E}=\frac{1-\delta_{1}^{2}}{E_{1}}+\frac{1-\delta_{2}^{2}}{E_{2}},$$

where *E*_*1*_ is the elastic modulus of coal (MPa), *E*_*2*_ is the elastic modulus of the middle plate (MPa), *δ*_1_ is the Poisson ratio of coal, *δ*_2_ is the Poisson ratio of the middle plate and *β* is the internal friction angle of coal.

*K*_1_, *K*_2_ and *ξ* are calculation parameters, as follows:

$$K_{1}=(1+\delta_{1})(\xi \tan^{-1}\xi -1)+\frac{3}{2}{\left(1+\xi^{2}\right)}^{-1};$$

$$K_{2}=(1+\delta_{1})(\xi \tan^{-1}\xi -1)+\frac{1}{2}{\left(1+\xi^{2}\right)}^{-1};\;\mathrm{and}$$

$$\xi =0.0475+(1+\delta_{1})(\frac{1-\sin\beta }{3+\sin\beta }).$$

Based on Eqs (1) and (4), the following can be obtained:

$$\Delta V=\frac{\pi }{3}h^{2}(3R-h)+[\frac{\pi R^{2}}{180}\mathrm{arc}\;\sin\frac{d}{2R}-\frac{d(R-h)}{2}]a$$

$$=f\{\left(m,R,v,\alpha ,\mu )(E_{1},E_{2},\delta_{1},\delta_{2},k,\beta \right)\}$$ (5)

Based on Eq (5), the pit volume formed by the impact damage due to raw coal to the middle plate can be characterized by the function *f*. The first part of function *f* captures the basic characteristics of raw coal, and the second part captures the contact parameter between the raw coal and middle plate.

The raw coal impacts the middle trough plate, and the impact can make the surface material of the middle trough plate fall off. Suppose that the impact wear volume *dV* of the raw coal acting on the middle trough plate is directly proportional to the impact energy *dA*. Then, the following applies:

dV=ψηdA (6)

where *η* is the dimensionless impact wear rate, which represents the wear amount per unit impact, and *ψ* is the impact energy conversion rate.

#### Finite element modeling

**Fig 1:**
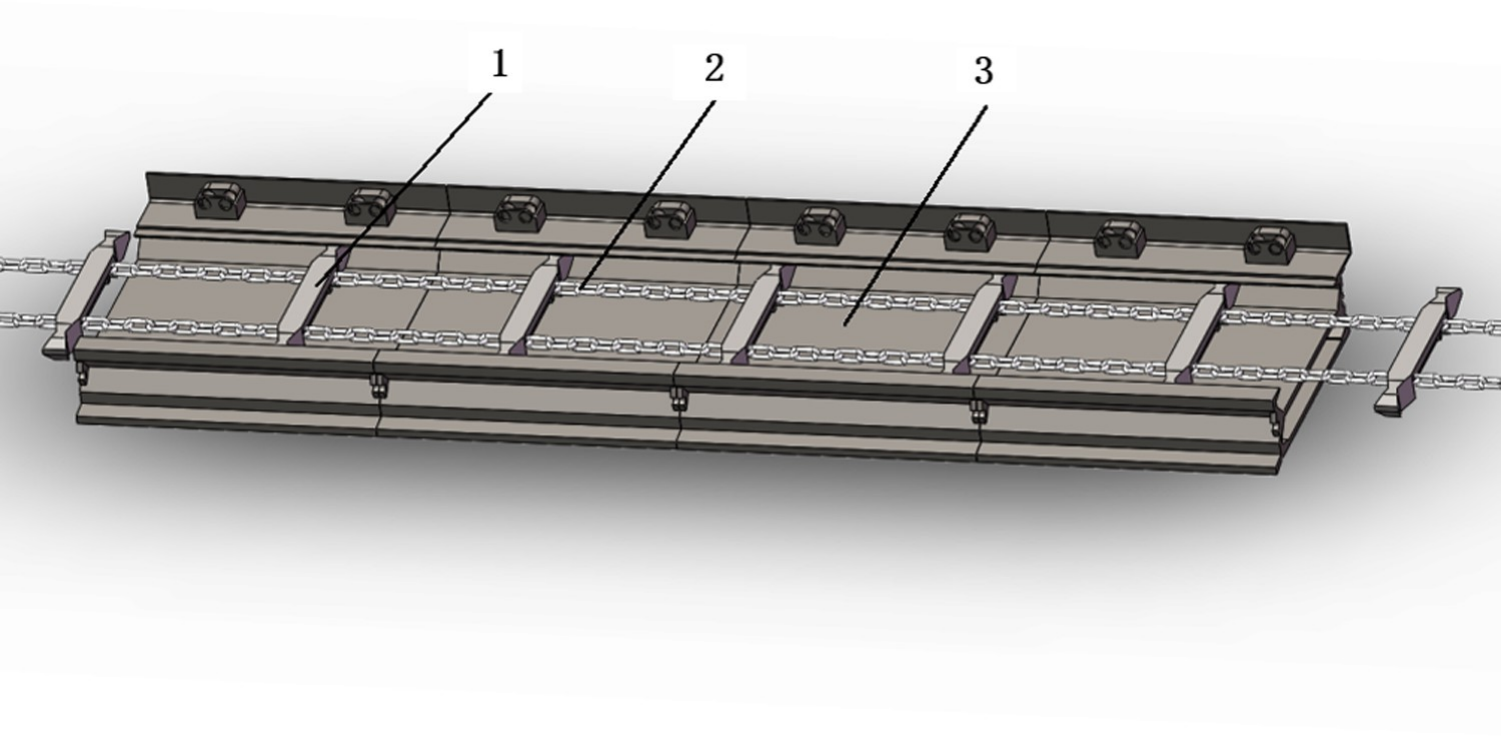
Assembly scheme of the middle trough of the scraper conveyor: 1-scraper, 2-chain, and 3-middle trough.

The simulation research object in this study is an 830-series scraper conveyor. Its conveying capacity is 1000 t/h, with a conveyor length of 200 m. This scraper conveyor adopts double-chain scraper chains with a scraper spacing of 108 mm, a running speed of 1.0 m/s, and a middle trough width of 780 mm. The length of a single segment of the middle trough is 1.5 m, with five segments in total; that is, the total length of the middle trough is 7.5 m. The installed power of the conveyor is 2×(250–315) kW. Fig 1 shows the assembly scheme of the middle trough and scraper chains.

**Fig 2:**
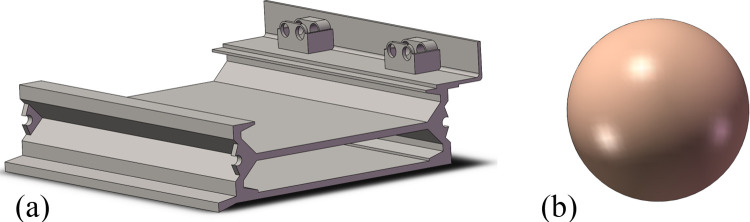
Solid model of the middle trough (a) and raw coal sphere model (b).

**Fig 3:**
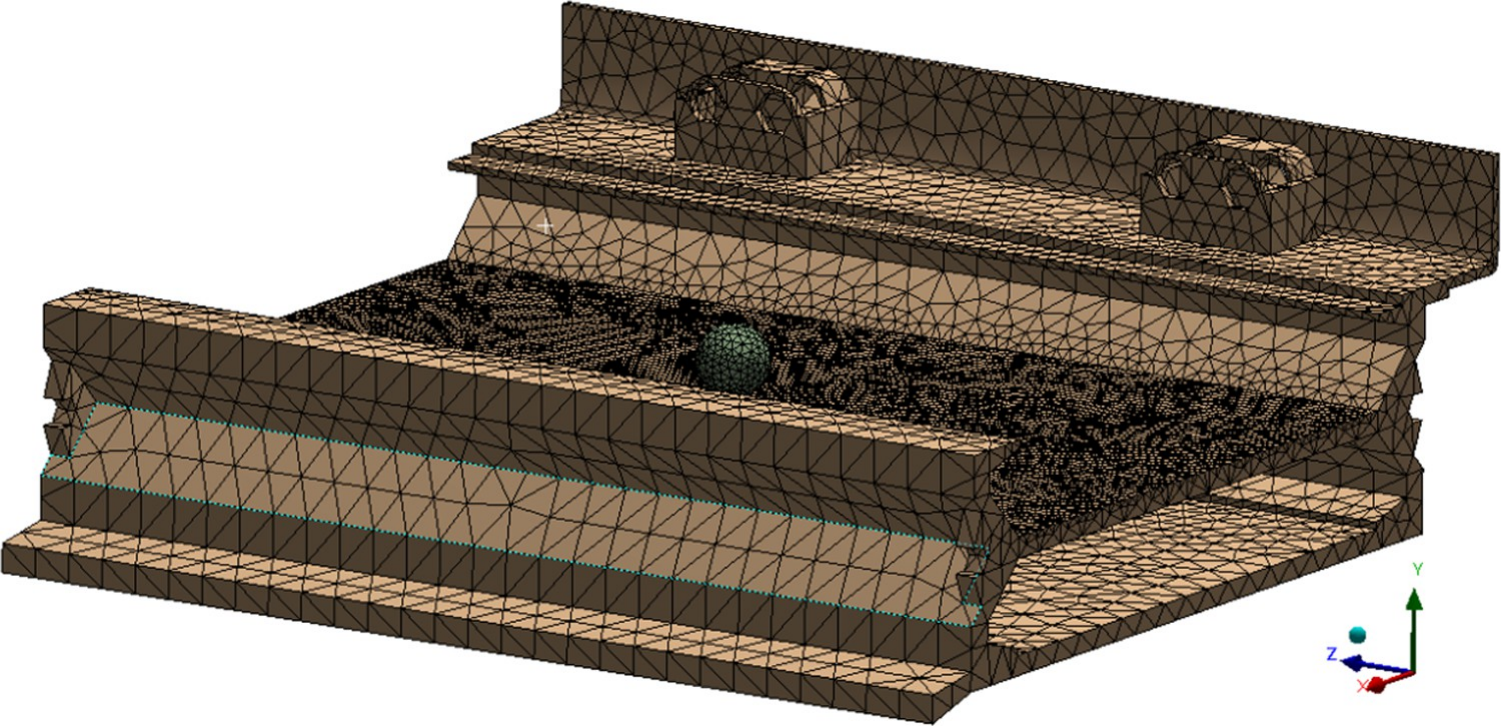
Grid model of a single section of the middle trough.

The raw coal is discretized to study the whole dynamic process of the impact damage due to raw coal to the middle trough of the scraper conveyor. Here, we suppose that when raw coal particles impact the middle plate of the middle trough, the contact between the raw coal particles and middle plate is subject to a point contact. Based on this supposition, spheres are used to replace raw coal particles to produce impact with the middle plate [23]. A particle impact model (Fig 2) is established in SolidWorks software (Dassault Systèmes, France), and the model is stored as an X_T file. The file is then introduced into the Explicit Dynamics module of ANSYS. In the model, single segments of the middle trough are treated as impacted objects with small balls as impacting objects. To ensure the accuracy of the simulation results and reduce the amount of computation, Hypermesh software is used to mesh the segment of the middle trough. Free meshing for tetrahedral solid elements is adopted. The unit length is set at 10 mm, with the minimal unit length at 2 mm, and the characteristic angle is set at 30°. A total of 1,283,942 units and 337,150 nodes are produced (Fig 3).

To complete simulation in EDEM software, the material property parameters of the middle trough and raw coal impact spheres (Table 4) as well as the properties of the contact between these objects must be set [24, 25] (Table 5).

**Table 4:** Material properties of the middle trough and raw coal impact spheres.

Material	Raw coal	Middle trough
Intensity (kg/m^3^)	1540	7800
Young’s modulus (GPa)	8.996	2000
Poisson’s ratio	0.3	0.3
Elastic modulus (GPa)	7.4967	1666.7
Shear modulus (GPa)	3.46	769.23
Specific heat (J/(kg·°C))	1220	434
Initial yield stress (MPa)	28	320

**Table 5:** Properties of the material contact.

Contact type	Static friction coefficient	Dynamic friction coefficient	Restitution coefficient
Coal-coal	0.4	0.06	0.5
Coal-steel	0.35	0.05	0.5

Regarding the particle plant created during EDEM simulation, the particle generation rate should meet the production rate of the 830-series scraper conveyor studied in this study, i.e., 1000 t/h. The speed of the generated raw coal particles along the moving direction of the scraper is 0.15 m/s, the rate of raw coal particle generation is 277 kg/s with a total weight of 1500 kg, and the simulation time step is set to 20% (50.15602 s) with a total simulation time of 5 s and a storage time interval of 0.1 s [26]. The distance between the impacting raw coal spheres and the middle trough is 0.001 mm, with a speed of the impacting spheres of 4.47 m/s [27, 28]. The simulation boundary conditions are set as follows: with the fixed simulation model and constant falling height and speed of materials, the falling height of the raw coal particles is 1 m, the running speed of the scraper chain is 1.0 m/s, the scraper spacing is 108 mm, and the flow of raw coal is 277 kg/s.

### Validation experiment

**Fig 4:**
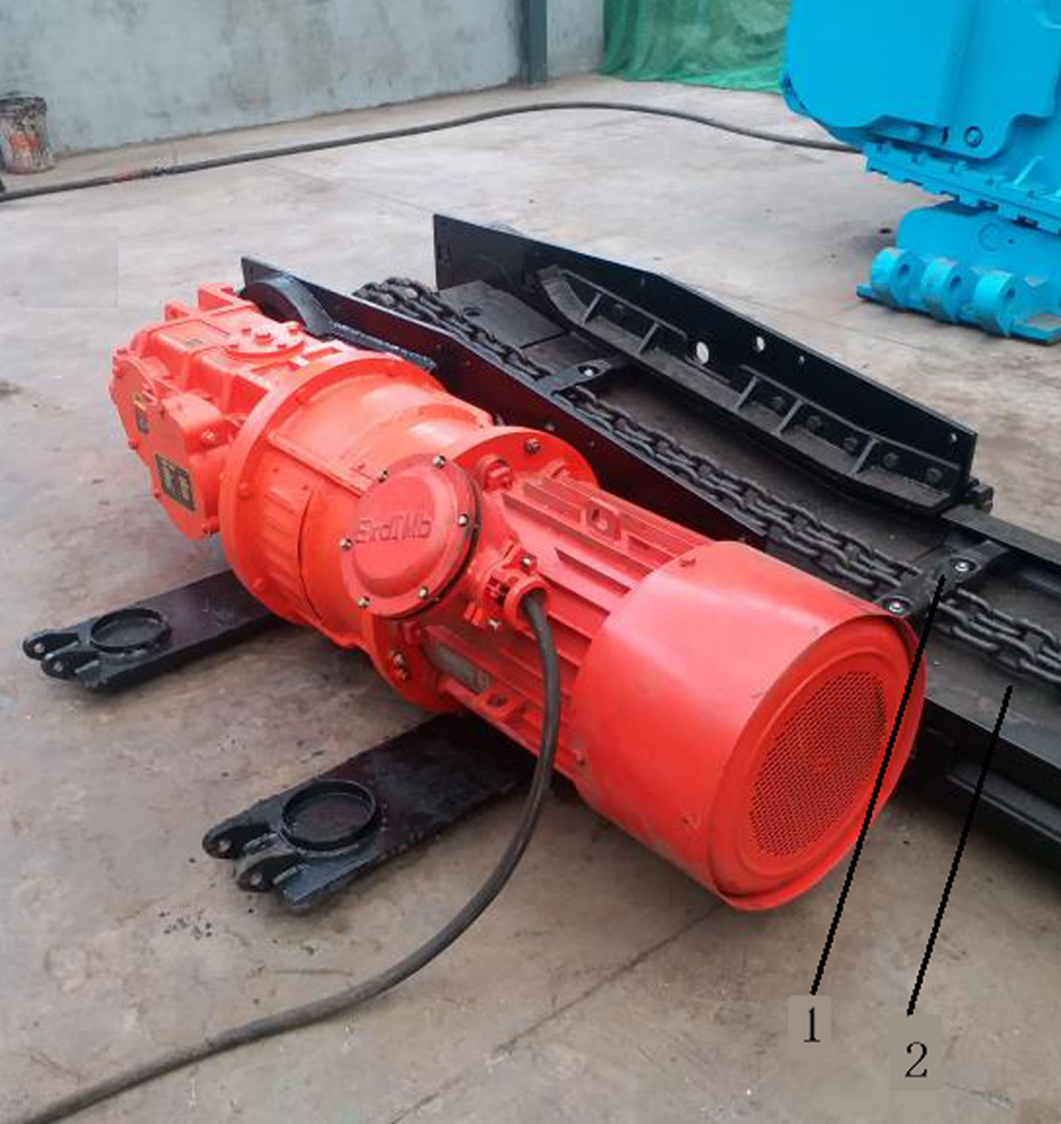
Scraper conveyor in the validation test: 1-scraper, 2-middle trough.

To verify the accuracy of the results obtained via OMA, we conducted an experiment. A scraper conveyor at a coal mine in Shanxi was tested by the Taiyuan Research Institute Co., Ltd., of the China Coal Science and Industry Group. The test equipment is shown in Fig 4. A power supply coil and lithium battery were set inside the scraper, and induction coils were set inside the middle plate of the trough. While the scraper conveyor was running, the scraper moved along the middle trough. When the scraper with an embedded power supply coil passed through the middle plate embedded with an induction coil, a voltage was generated at both ends of the induction coil. The voltage signal was collected by an acquisition module and transmitted to an industrial control computer. With increasing wear of the middle plate of the trough, the wear amount of the middle plate was calculated by an industrial control computer [29].

## Results and discussion

Within the context of explicit dynamics, research on impact can be performed only with a model under transient conditions. Therefore, to simplify the falling process of raw coal particles, the spacing between the impacting spheres and the middle trough is set to 0.001 mm, and the velocity is set to 4.470 m/s in this study.

### Simulation results

**Fig 5:**
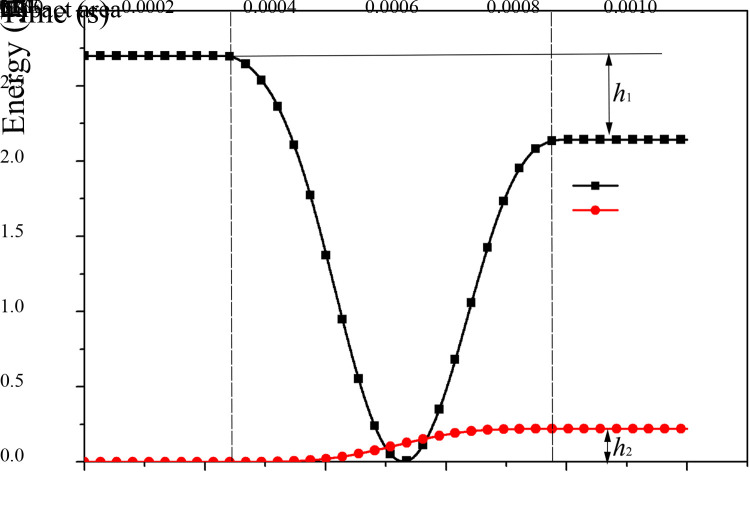
Curve of the impact energy. CKE, coal kinetic energy. MTE, energy of the middle trough.

Based on ANSYS simulation software, curves of the middle trough energy and the kinetic energy of raw coal impacting particles during the impact process are obtained (Fig 5).

In the impact area, the velocity of the middle plate reaches a maximum value of 112.39 mm/s. From the beginning of middle plate impact to rebound, the kinetic energy of the coal particle spheres first decreases and then increases while the energy of the middle plate and trough sides gradually increases. During the impact process, the energy of the middle trough increases by *h*_*2*,_ and the kinetic energy of the raw coal impact particles decreases by *h*_*1*_. At this moment, the conversion rate of the impact energy according to impact theory is calculated as follows:

$$\psi =\frac{h_{2}}{h_{1}}\times 100\%$$ (7)

Based on the simulation results, *h*_1_ = 2.699–2.141 = 0.558 and *h*_2_ = 0.221, *ψ* = 39.6% are obtained.

A correction is made according to the above finite element simulation equation (Eq (6)) as follows:

dV=0.396ηdW

Based on the ANSYS simulation, from falling to landing on the middle trough, approximately 39% of the dynamic energy of raw coal is absorbed by the middle trough and transformed into impact energy. Grincova et al. [5] monitored the energy absorbed by the conveyor belt at the material receiving place of the general belt conveyor and found that the impact damage to the conveyor belt is associated with the dynamic energy loss of the falling materials.

**Fig 6:**
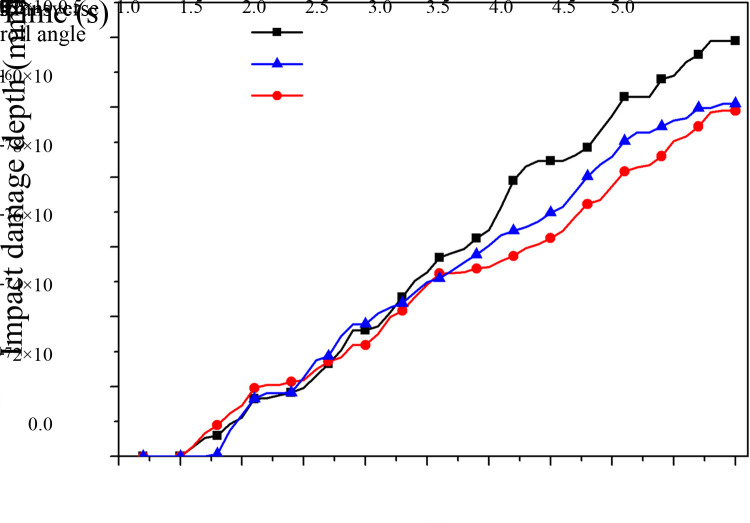
Impact damage depth curve of the middle trough under the different transverse roll angles.

**Fig 7:**
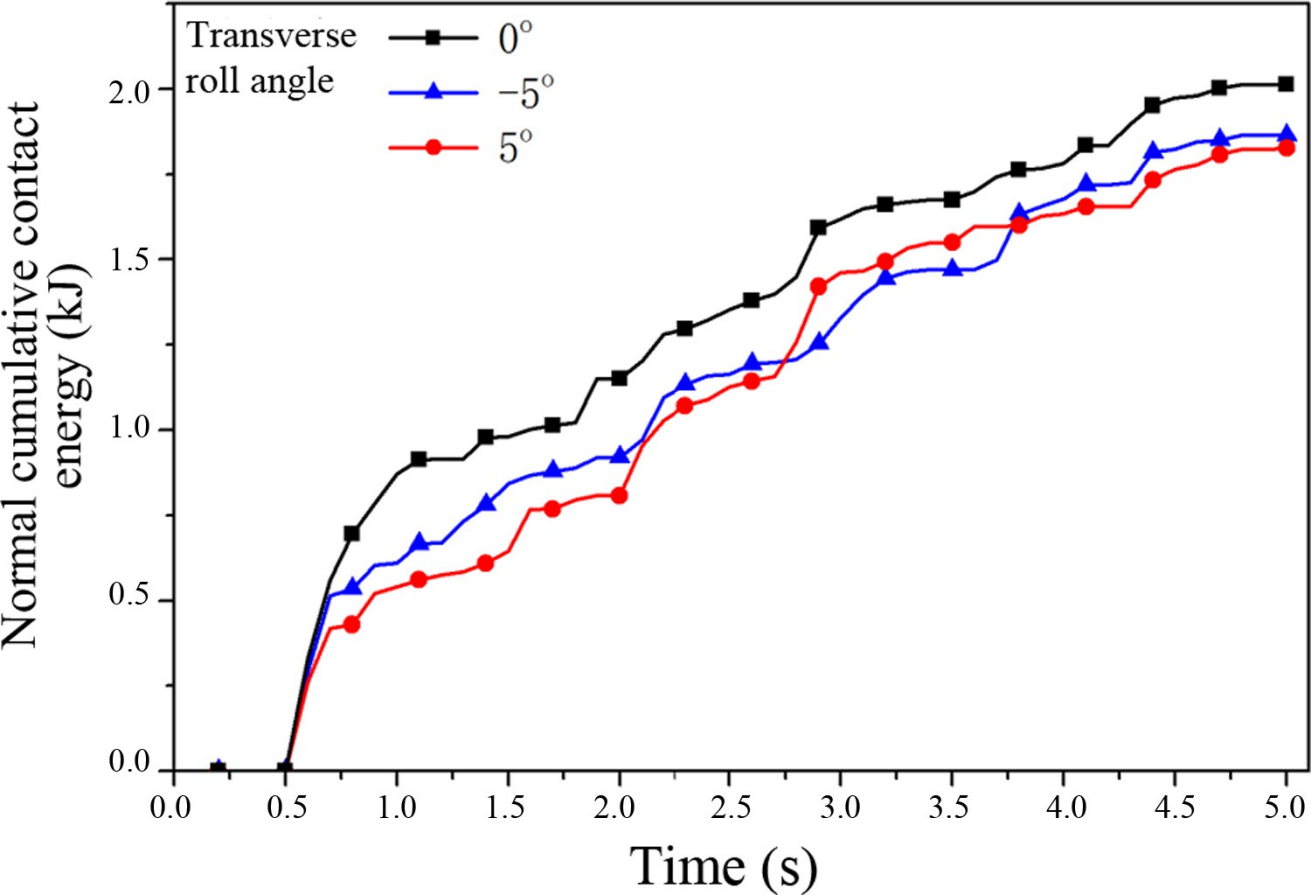
Normal cumulative contact energy curve of the middle trough under the different transverse roll angles.

In EDEM software, the impact of raw coal on the middle trough under the conditions of the four factors at the three levels is simulated separately. An impact damage depth curve of raw coal on the middle groove under different transverse roll angles is obtained (Fig 6), and the normal cumulative contact energy curve is shown in Fig 7.

As shown in Figs 6 and 7, the process of raw coal falling occurs before the time point of 0.5 s. Between 0.5 s and 5 s, the average impact damage depth and normal cumulative contact energy of the middle trough reach maximum values when the transverse roll angle is 0°, followed by -5° and then 5° (Table 6). The simulation results indicate that when there is a transverse roll angle of the middle trough, the load components of the normal impact of raw coal falling onto the middle trough change, and the middle trough is mainly affected by the normal impact load of the raw coal. Therefore, the occurrence of a transverse roll angle of the middle trough results in changes in the average impact damage depth and cumulative contact energy of the middle plate. In addition, as shown in Table 6, when there is a transverse roll angle of the middle trough, particularly with a positive value, the angle causes the load of raw coal to fall onto the middle trough to produce components, which reduces the contact load on the middle trough, thereby alleviating the impact damage to the middle trough. This finding is basically consistent with that reported in the literature [16], according to which an increase in contact pressure aggravates the wear of the middle trough. When the transverse roll angle is 0°, the contact load of falling coal on the middle trough is increased, as no components are produced, which leads to the most severe damage to the middle trough.

**Table 6:** Simulation results of the impact damage to the middle plate under the different transverse roll angles.

Transverse roll angle (°)	Average impact damage depth (mm)	Normal accumulative contact energy (kJ)
0	1.188×10^−6^	2.013
-5	1.039×10^−6^	1.863
5	0.99×10^−6^	1.826

**Fig 8:**
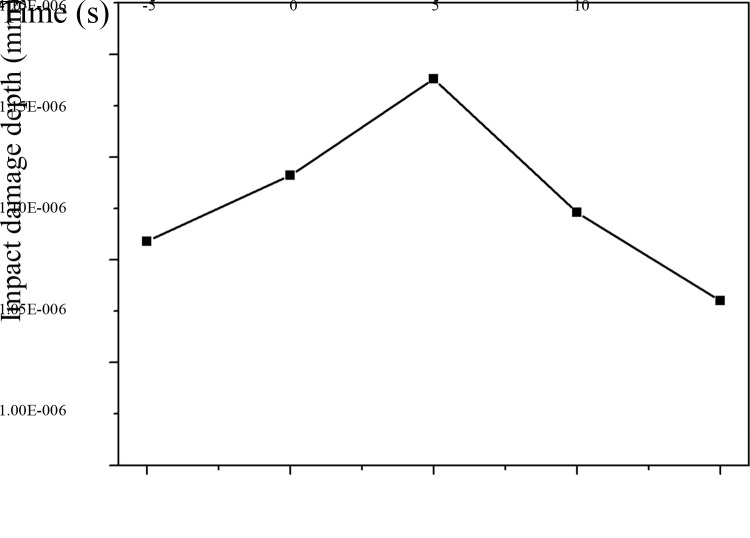
Impact damage depth curve of the middle trough under the different front lean angles.

**Fig 9:**
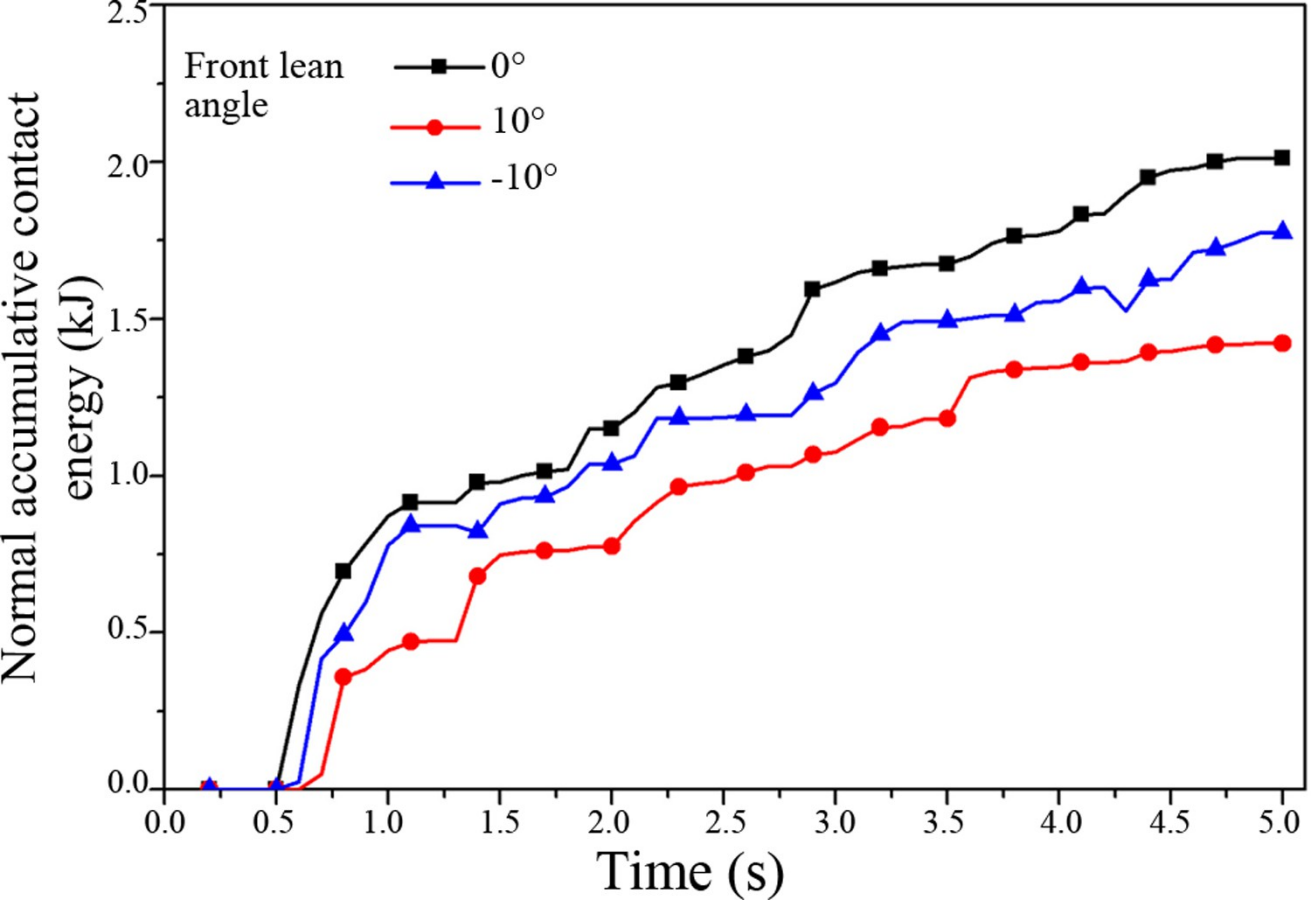
Normal accumulative contact energy curve of the middle trough under the different front lean angles.

Considering the different front lean angles, the scraper conveyor is operated under different transportation conditions, such as upward, downward and horizontal transportation. Impact damage depth and normal cumulative contact energy curves are shown in Figs 8 and 9, respectively.

When the front lean angle is positive (i.e., 10°), the scraper conveyor occurs in the upward transportation state. The falling of raw coal onto the middle trough generates a velocity component opposite to that of the scraper chain. The reduction in velocity reduces the kinetic energy of raw coal and minimizes the impact on the middle trough and the normal cumulative contact energy. When the front lean angle is negative (-10°), the scraper conveyor occurs in the downward transportation state. The falling of raw coal onto the middle trough generates a velocity component consistent with that of the scraper chain. Superposition of the velocity increases the kinetic energy of the raw coal, leading to a great impact on the middle trough. At this moment, the normal cumulative contact energy is also high. When the front lean angle is 0°, the scraper conveyor occurs in the horizontal transportation state. The falling of raw coal directly impacts the middle trough. There is no velocity component of up- or downward transportation. All kinetic energy acts on the middle trough, resulting in the maximum impact upon the middle trough. The normal cumulative contact energy also reaches a maximum value. Our findings are consistent with the result reported by Sinha and Mukhopadhyay [16]. The results are summarized in Table 7.

**Table 7:** Simulation results of the impact damage to the middle plate under the different front lean angles.

Front lean angle (°)	Average impact damage depth (mm)	Normal accumulative contact energy (kJ)
0	1.231×10^−6^	2.013
-10	1.113×10^−6^	1.774
10	1.065×10^−6^	1.421

**Fig 10:**
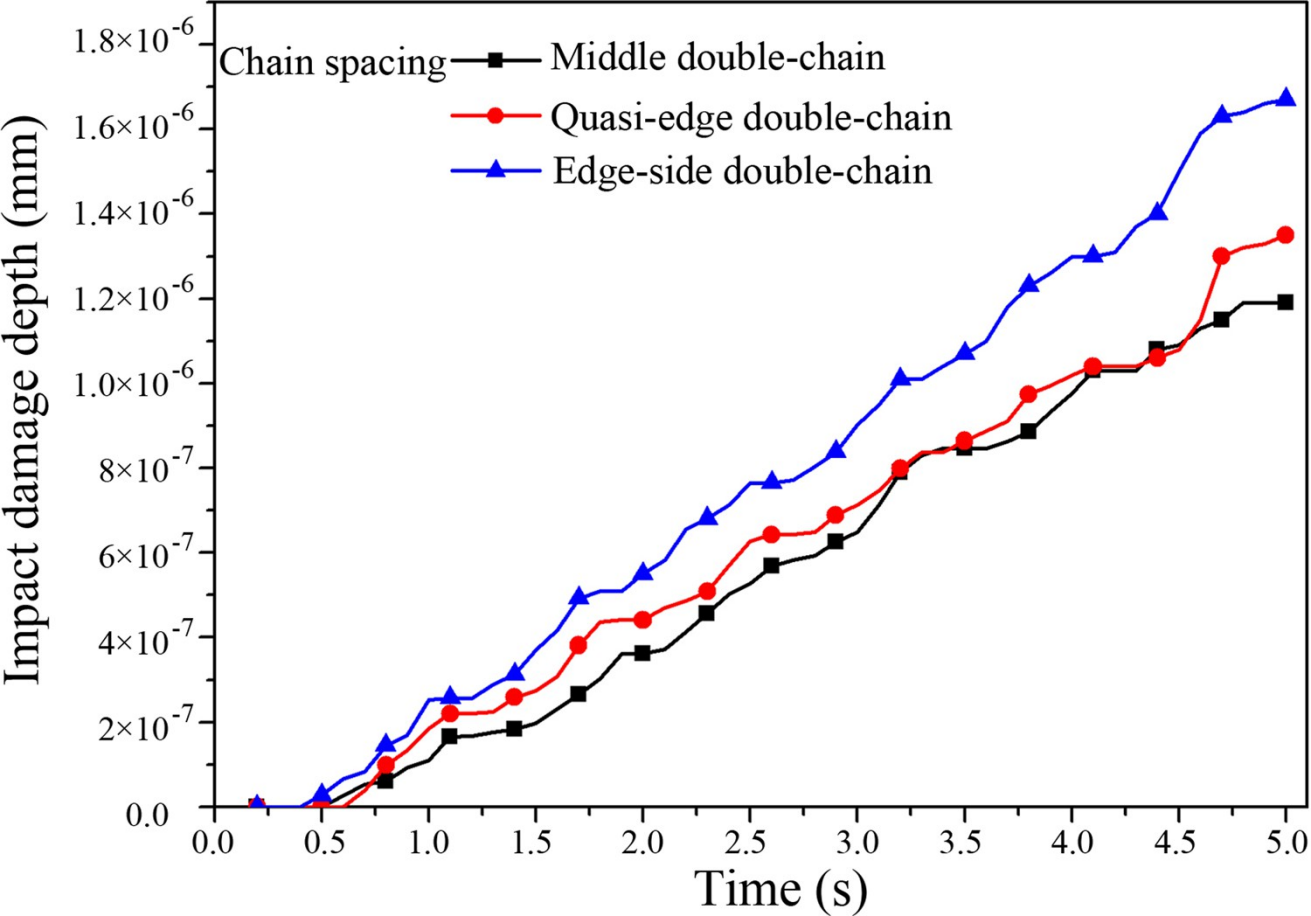
Impact damage depth curve of the middle trough under the different chain spacings.

**Fig 11:**
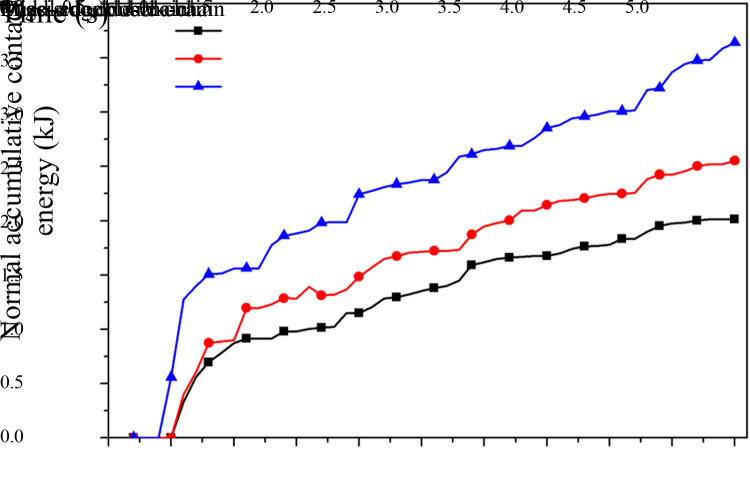
Normal accumulative contact energy curve of the middle trough under the different chain spacings.

Impact damage depth and normal accumulative contact energy curves of the middle trough under the different chain spaces are shown in Figs 10 and 11, respectively. When the chain layout and spacing differ, the contact area between the raw coal and middle plate also changes. According to the analysis of Figs 10 and 11, under the edge-side double-chain layout, the average impact damage depth of the middle plate is the deepest, and the cumulative normal contact energy is the highest, followed by the quasi-edge double-chain and then middle double-chain layouts. With different chain layouts, the wear of the middle trough of the scraper conveyor also differs, and therefore, the selection of the chain layout plays a critical role in the design of a scraper conveyor [10]. Our results are summarized in Table 8.

**Table 8:** Simulation results of the impact damage under the different chain spacings.

Chain spacing (mm)	Average impact damage depth (mm)	Normal accumulative contact energy (kJ)
700	1.673×10^−6^	3.639
420	1.354×10^−6^	2.552
140	1.188×10^−6^	2.013

**Fig 12:**
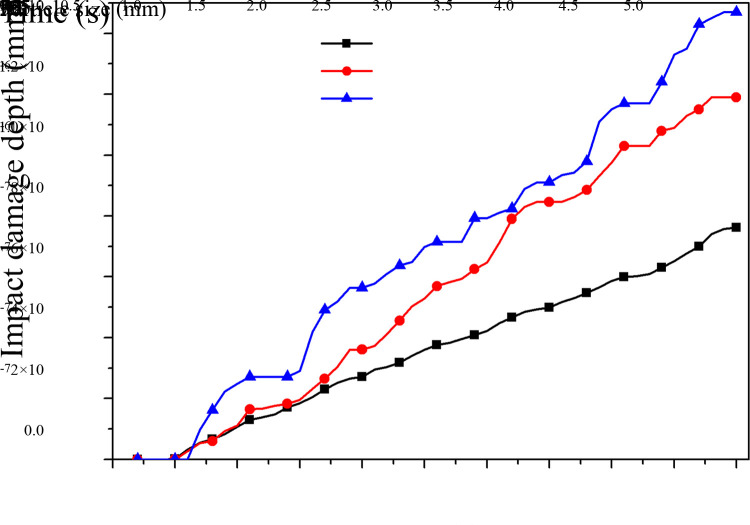
Curve of the impact damage depth of the middle trough caused by raw coal with different particle sizes.

**Fig 13:**
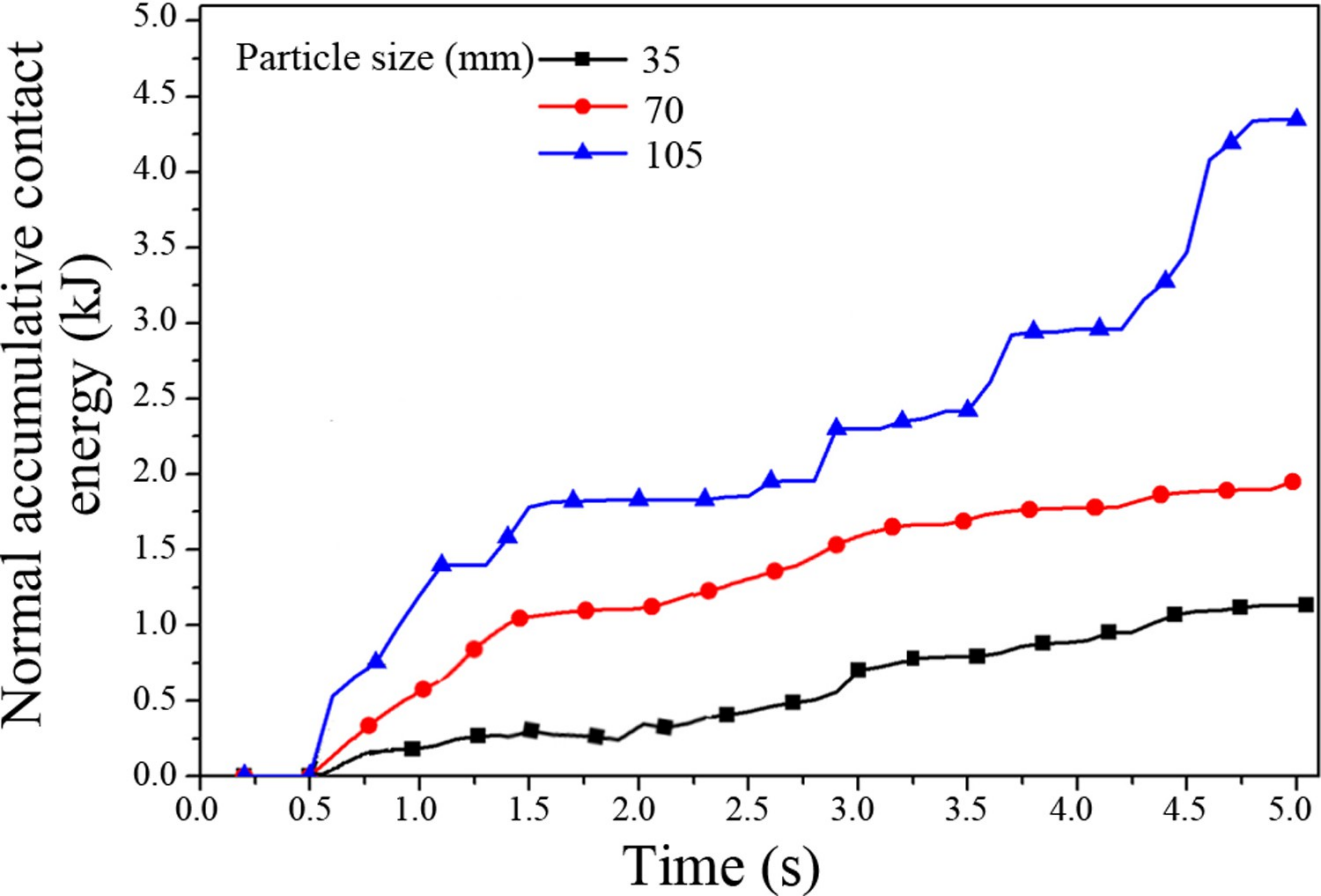
Curve of the normal accumulative contact energy of the middle trough caused by raw coal with different particle sizes.

Impact damage depth and normal accumulative contact energy curves of the middle trough for the different coal particle sizes are shown in Figs 12 and 13, respectively. The larger the particle size of raw coal is, the deeper the average impact damage depth of the plate and the higher the normal cumulative contact energy. Shi and Zhu [3] and Glushkova [4] investigated the influence of the particle size and moisture content of raw coal on the damage to the middle trough, and they found that a larger particle size leads to more prominent damage to the middle trough.

### Orthogonal results

Based on the simulation results related to the four factors at the three levels, as summarized in Tables 1 and 6–9, the impact damage depth and normal accumulative contact energy of the middle trough under the different conditions are obtained (Tables 10 and 11, respectively; S1 File).

**Table 9:** Simulation results of the impact damage caused by the different sizes of raw coal particles.

Raw coal particle size (mm)	Average impact damage depth (mm)	Normal accumulative contact energy (kJ)
35	0.7619×10^−6^	1.066
70	1.188×10^−6^	2.013
105	1.467×10^−6^	4.347

**Table 10:** Impact damage depth (mm) under the different conditions.

Parameter	Transverse roll angle (°)	Front lean angle (°)	Particle size (mm)	Chain spacing (mm)
*P*_11_	3.998×10^−6^	3.996×10^−6^	2.736×10^−6^	3.305×10^−6^
*P*_12_	3.903×10^−6^	3.987×10^−6^	4.109×10^−6^	3.675×10^−6^
*P*_13_	3.908×10^−6^	3.826×10^−6^	4.964×10^−6^	4.829×10^−6^
${\bar{P}}_{11}$	1.333×10^−6^	1.332×10^−6^	0.912×10^−6^	1.098×10^−6^
${\bar{P}}_{12}$	1.301×10^−6^	1.329×10^−6^	1.366×10^−6^	1.225×10^−6^
${\bar{P}}_{13}$	1.299×10^−6^	1.272×10^−6^	1.655×10^−6^	1.610×10^−6^

**Table 11:** Normal accumulative contact energy (kJ) under the different conditions.

Parameter	Transverse roll angle (°)	Front lean angle (°)	Particle size (mm)	Chain spacing (mm)
*P*_21_	8.515	7.825	4.261	5.702
*P*_22_	7.726	7.72	8.307	6.866
*P*_23_	6.897	7.593	10.57	10.56
${\bar{P}}_{21}$	2.838	2.608	1.421	1.901
${\bar{P}}_{22}$	2.576	2.574	2.769	2.292
${\bar{P}}_{23}$	2.299	2.531	3.523	3.52

In the tables, *p*_1i_ denotes the sum of the impact damage depth under the four factors at the *i*th level, ${\bar{P}}_{1i}$ denotes the average of the impact damage depth under each factor at the *i*th level, *p*_2i_ denotes the sum of the accumulative contact energy under the four factors at the *i*th level, ${\bar{P}}_{2i}$ and denotes the average of the accumulative contact energy under each factor at the *i*th level.

Based on matrix numerical analysis [30], an impact damage depth matrix *P*_*1*_ and normal accumulative contact energy matrix *P*_*2*_ are obtained as follows:

$$P_{1}=[\begin{array}{cccc} 1.333 & 0 & 0 & 0\\ 1.301 & 0 & 0 & 0\\ 1.299 & 0 & 0 & 0\\ 0 & 1.332 & 0 & 0\\ 0 & 1.329 & 0 & 0\\ 0 & 1.272 & 0 & 0\\ 0 & 0 & 0.912 & 0\\ 0 & 0 & 1.366 & 0\\ 0 & 0 & 1.655 & 0\\ 0 & 0 & 0 & 1.098\\ 0 & 0 & 0 & 1.225\\ 0 & 0 & 0 & 1.610 \end{array}]\times 10^{-6}$$

$$P_{2}=[\begin{array}{cccc} 2.838 & 0 & 0 & 0\\ 2.576 & 0 & 0 & 0\\ 2.299 & 0 & 0 & 0\\ 0 & 2.608 & 0 & 0\\ 0 & 2.574 & 0 & 0\\ 0 & 2.531 & 0 & 0\\ 0 & 0 & 1.420 & 0\\ 0 & 0 & 2.769 & 0\\ 0 & 0 & 3.524 & 0\\ 0 & 0 & 0 & 1.901\\ 0 & 0 & 0 & 2.292\\ 0 & 0 & 0 & 3.520 \end{array}]$$

A factor layer matrix is obtained as follows:

$$W_{1}=[\begin{array}{cccc} \frac{1}{3.933} & 0 & 0 & 0\\ 0 & \frac{1}{3.933} & 0 & 0\\ 0 & 0 & \frac{1}{3.933} & 0\\ 0 & 0 & 0 & \frac{1}{3.933} \end{array}]\times 10^{6}$$

$$W_{2}=[\begin{array}{cccc} \frac{1}{7.713} & 0 & 0 & 0\\ 0 & \frac{1}{7.713} & 0 & 0\\ 0 & 0 & \frac{1}{7.713} & 0\\ 0 & 0 & 0 & \frac{1}{7.713} \end{array}]$$

A horizontal layer matrix is obtained as follows:

$$X_{1}=[\begin{array}{c} \frac{0.034}{1.349}\\ \frac{0.060}{1.349}\\ \frac{0.743}{1.349}\\ \frac{0.512}{1.349} \end{array}]$$

$$X_{2}=[\begin{array}{c} \frac{0.539}{4.335}\\ \frac{0.077}{4.335}\\ \frac{2.102}{4.335}\\ \frac{1.619}{4.335} \end{array}]$$

An evaluation index weight matrix is obtained as follows:

$$G_{1}=P_{1}W_{1}X_{1}=[\begin{array}{cccc} 1.333 & 0 & 0 & 0\\ 1.301 & 0 & 0 & 0\\ 1.299 & 0 & 0 & 0\\ 0 & 1.332 & 0 & 0\\ 0 & 1.329 & 0 & 0\\ 0 & 1.272 & 0 & 0\\ 0 & 0 & 0.912 & 0\\ 0 & 0 & 1.366 & 0\\ 0 & 0 & 1.655 & 0\\ 0 & 0 & 0 & 1.098\\ 0 & 0 & 0 & 1.225\\ 0 & 0 & 0 & 1.610 \end{array}]\times 10^{-6}\times [\begin{array}{cccc} \frac{1}{3.933} & 0 & 0 & 0\\ 0 & \frac{1}{3.933} & 0 & 0\\ 0 & 0 & \frac{1}{3.933} & 0\\ 0 & 0 & 0 & \frac{1}{3.933} \end{array}]\times 10^{-6}\times [\begin{array}{c} \frac{0.034}{1.349}\\ \frac{0.060}{1.349}\\ \frac{0.743}{1.349}\\ \frac{0.512}{1.349} \end{array}]=[\begin{array}{c} 0.0085\\ 0.0083\\ 0.0083\\ 0.0151\\ 0.0150\\ 0.0144\\ 0.1277\\ 0.1913\\ 0.2318\\ 0.1060\\ 0.1182\\ 0.1554 \end{array}]$$

$$G_{2}=P_{2}W_{2}X_{2}=[\begin{array}{cccc} 2.838 & 0 & 0 & 0\\ 2.576 & 0 & 0 & 0\\ 2.299 & 0 & 0 & 0\\ 0 & 2.608 & 0 & 0\\ 0 & 2.574 & 0 & 0\\ 0 & 2.531 & 0 & 0\\ 0 & 0 & 1.420 & 0\\ 0 & 0 & 2.769 & 0\\ 0 & 0 & 3.524 & 0\\ 0 & 0 & 0 & 1.901\\ 0 & 0 & 0 & 2.292\\ 0 & 0 & 0 & 3.520 \end{array}]\times [\begin{array}{cccc} \frac{1}{7.713} & 0 & 0 & 0\\ 0 & \frac{1}{7.713} & 0 & 0\\ 0 & 0 & \frac{1}{7.713} & 0\\ 0 & 0 & 0 & \frac{1}{7.713} \end{array}]\times [\begin{array}{c} \frac{0.539}{4.335}\\ \frac{0.077}{4.335}\\ \frac{2.102}{4.335}\\ \frac{1.619}{4.335} \end{array}]=[\begin{array}{c} 0.0457\\ 0.0415\\ 0.0370\\ 0.0060\\ 0.0059\\ 0.0058\\ 0.0892\\ 0.1740\\ 0.2215\\ 0.0920\\ 0.1110\\ 0.1704 \end{array}]$$

Averages of the impact damage depth evaluation index weight matrix *G*_*1*_ and the normal accumulative contact energy evaluation index weight matrix *G*_*2*_ are calculated, and a total weight matrix is obtained as follows:

$$G=\frac{G_{1}+G_{2}}{2}=\frac{1}{2}\times [\begin{array}{c} 0.0085\\ 0.0083\\ 0.0083\\ 0.0151\\ 0.0150\\ 0.0144\\ 0.1277\\ 0.1913\\ 0.2318\\ 0.1060\\ 0.1182\\ 0.1554 \end{array}+\begin{array}{c} 0.0457\\ 0.0415\\ 0.0370\\ 0.0060\\ 0.0059\\ 0.0058\\ 0.0892\\ 0.1740\\ 0.2215\\ 0.0920\\ 0.1110\\ 0.1704 \end{array}]=[\begin{array}{c} 0.0271\\ 0.0249\\ 0.0227\\ 0.0106\\ 0.0104\\ 0.0101\\ 0.1084\\ 0.1827\\ 0.2266\\ 0.0990\\ 0.1146\\ 0.1629 \end{array}]=[\begin{array}{c} A_{1}\\ A_{2}\\ A_{3}\\ B_{1}\\ B_{2}\\ B_{3}\\ C_{1}\\ C_{2}\\ C_{3}\\ D_{1}\\ D_{2}\\ D_{3} \end{array}]$$

Based on the weight coefficient, the weight of each factor in impact damage to the middle trough is determined:

$$U=\sum_{i=1}^{3}u_{i}$$

where *U* denotes the weight coefficient of the influencing factor and *u* denotes the value of the evaluation index weight matrix parameter (here, A, B, C and D).

Data are introduced, and the following values are obtained:

$$U_{A}=0.0747$$

$$U_{B}=0.0311$$

$$U_{C}=0.5177$$

$$U_{D}=0.3765$$

**Fig 14:**
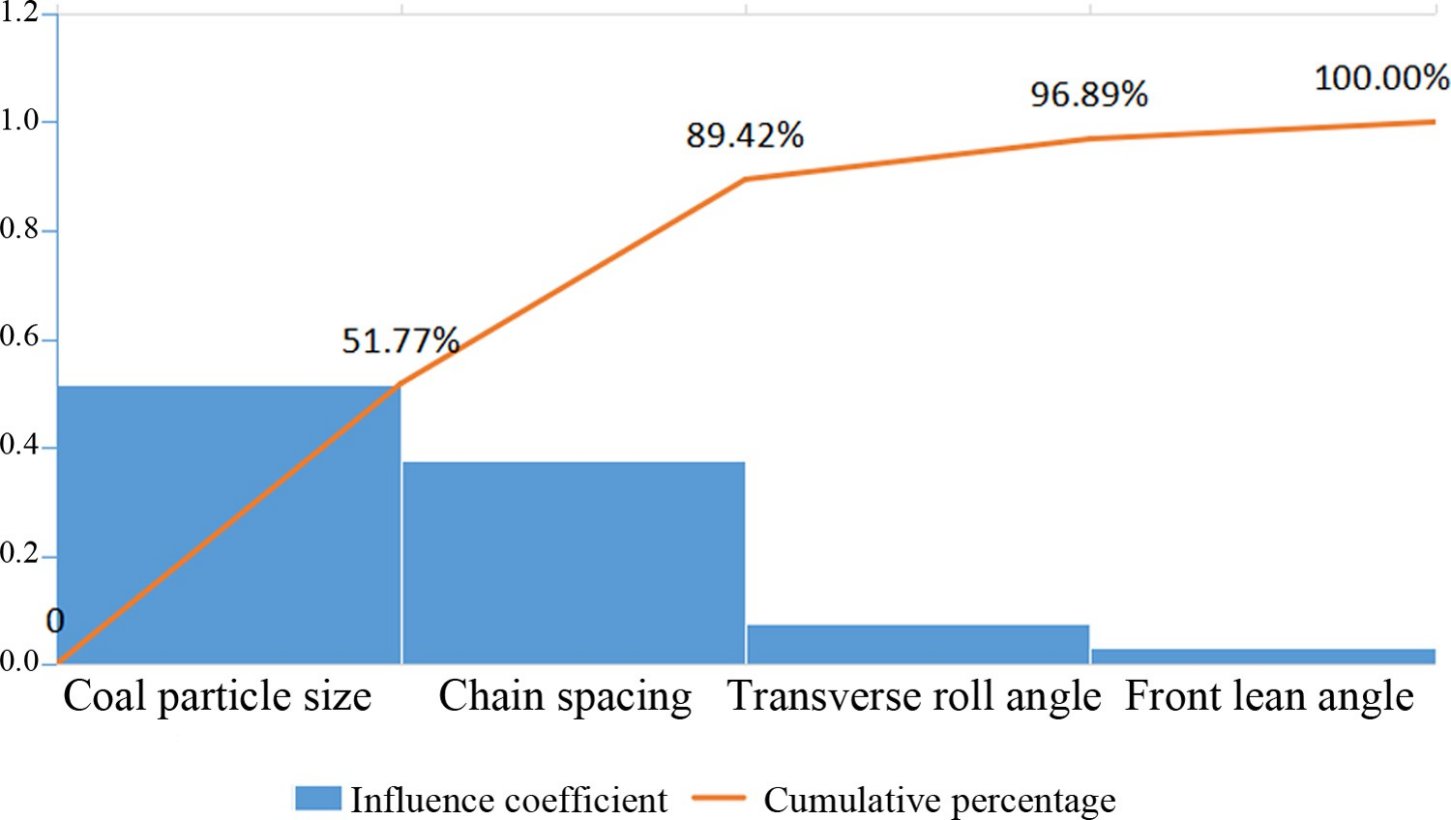
Pareto diagram of the impact damage factors of the middle trough.

The smaller the impact damage depth and normal cumulative contact energy are, the better the effect. According to matrix numerical analysis, the influence of the four factors on the evaluation indices of the impact damage depth and normal cumulative contact energy can be ordered as C > D > A > B, namely, raw coal particle size > chain spacing > roll angle > front lean angle. The Pareto diagram of the impact damage factors of the middle trough is obtained from the weight coefficients of the influencing factors (Fig 14).

Based on Tables 4 and 5 as well as the total weight matrix *G*, the three horizontal conditions of the four factors affecting impact damage to the middle trough can be ordered as A_3_ < A_2_ <A_1_, B_3_ < B_2_ < B_1_, C_1_ < C_2_ < C_3_, and D_1_ < D_2_ < D_3_. By combining the levels with the least impact on the impact damage depth and normal cumulative contact energy, the optimal combination design is A_3_B_3_C_1_D_1_, i.e., the roll angle is 5°, the front lean angle is 10°, the chain spacing is 140 mm, and the particle size is 0.5 times the basic particle size. Under these conditions, the impact damage to the middle plate of the scraper conveyor is the lowest.

### Experimental results

Based on the test combination of the four factors and three levels, the impact wear on the middle trough under the corresponding horizontal values of the four influencing factors is obtained through the test of the scraper conveyor, as shown in Fig 4. The experimental results are listed in Table 12.

**Table 12:** Wear amount of the middle trough in the validation experiment.

Parameter	Wear amount (ng/mm)			
	A	B	C	D
	Transverse roll angle (°)	Front lean angle (°)	Particle size (mm)	Chain spacing (mm)
${\bar{P}}_{11}$	8.1100	9.0630	6.2052	6.6802
${\bar{P}}_{12}$	7.9153	9.0425	9.2943	7.4529
${\bar{P}}_{13}$	7.9031	8.6547	11.2606	9.7952

As revealed in Table 12, A_3_ < A_2_ < A_1_, B_3_ < B_2_ < B_1_, C_1_ < C_2_ < C_3_ and D_1_ < D_2_ < D_3_. The experimental results also demonstrate that the optimal working condition combination is A_3_B_3_C_1_D_1_, consistent with the results of the simulation experiment.

The correlations of the wear amount of the middle trough measured in the experiment with the normal cumulative contact energy and impact damage depth obtained in the OMA simulation are analyzed using Pearson analysis, and the results are summarized in Tables 13 and 14, respectively.

**Table 13:** Correlation of the wear amount of the middle trough measured in the experiment with the normal cumulative contact energy obtained in the OMA simulation.

Item	Parameter	A	B	C	D
Wear amount (ng/mm)	${\bar{P}}_{11}$	8.1100	9.0630	6.2052	6.6802
	${\bar{P}}_{12}$	7.9153	9.0425	9.2943	7.4529
	${\bar{P}}_{13}$	7.9031	8.6547	11.2606	9.7952
Normal cumulative contact energy (kJ)	${\bar{P}}_{21}$	2.838	2.608	1.421	1.901
	${\bar{P}}_{22}$	2.576	2.574	2.769	2.292
	${\bar{P}}_{23}$	2.299	2.531	3.523	3.52
Correlation coefficient (r)		0.8837	0.9165	0.9994	0.9999
P value		0.0310	0.0262	0.0223	0.0043

Notes: Correlation analysis is performed using the Pearson method. A, transverse roll angle (°); B, front lean angle (°); C, particle size (mm); D, chain spacing (mm).

**Table 14:** Correlation of the wear amount of the middle trough measured in the experiment with the impact damage depth obtained in the OMA simulation.

Item	Parameter	A	B	C	D
Wear amount (ng/mm)	${\bar{P}}_{11}$	8.1100	9.0630	6.2052	6.6802
	${\bar{P}}_{12}$	7.9153	9.0425	9.2943	7.4529
	${\bar{P}}_{13}$	7.9031	8.6547	11.2606	9.7952
Impact damage depth (mm)	${\bar{P}}_{21}$	1.333×10^−6^	1.332×10^−6^	0.912×10^−6^	1.098×10^−6^
	${\bar{P}}_{22}$	1.301×10^−6^	1.329×10^−6^	1.366×10^−6^	1.225×10^−6^
	${\bar{P}}_{23}$	1.299×10^−6^	1.272×10^−6^	1.655×10^−6^	1.610×10^−6^
Correlation coefficient (r)		0.9999	0.9999	0.9999	0.9999
P value		0.033	0.003	0.003	0.003

Notes: Correlation analysis is performed using the Pearson method. A, transverse roll angle (°); B, front lean angle (°); C, particle size (mm); D, chain spacing (mm).

The wear amount of the middle trough obtained in the experiment has a complete positive correlation with the impact damage depth and a positive correlation with the normal cumulative contact energy obtained by the OMA method. The normal cumulative contact energy obtained by the OMA method is correlated with the transverse roll angle, front lean angle and particle size at a significance level of 0.05 and with the chain spacing at a significance level of 0.01 in the verification experiment. The impact damage depth obtained by the OMA method is correlated with the transverse roll angle at a significance level of 0.05 and with the front lean angle, particle size and chain spacing at a significance level of 0.01 in the verification experiment. The experimental results are consistent with those obtained using EDEM simulation and OMA; that is, the impact damage of the middle trough is the smallest when the raw coal particle size is the smallest, the chain is arranged with medium double chains, and the transverse roll angle and front lean angle of the middle groove are positive. The results of this study are consistent with those reported in the literature [21].

## Conclusions

Impact damage to the middle trough of a scraper conveyor is the consequence of the combined action of a variety of factors. At present, no effective method to quickly determine the impact damage factors of scraper conveyors has been reported. The purpose of this paper is to establish a four-factor, three-level orthogonal test table using the orthogonal matrix analysis method, and the impact damage depth and normal accumulative contact energy of the middle trough corresponding to each factor at each level are obtained via EDEM simulations. Based on matrix analysis, the optimal working condition combination to minimize impact damage to the middle trough of a scraper conveyor is established. This study provides a method to effectively reduce the number of experiments and thus to efficiently determine the optimal working condition combination for scraper conveyors. The main findings of this study are as follows.

1. According to the obtained weight coefficient, the raw coal particle size yields the greatest influence on the impact damage to the middle trough, followed by the chain spacing transverse roll angle and front lean angle;
2. Through OMA and actual measurements, the optimal combination of working conditions is obtained as follows: a roll angle of 5°, front lean angle of 10°, minimum particle size and double-chain layout spacing of 140 mm.

This study suffers from limitations. Due to the complexity and particularity of underground operation in coal mines, scraper conveyor experiments can be simulated and tested only before a scraper conveyor traverses down the mine. Therefore, there remains a large gap between the test and underground field operation conditions, which results in limited experimental result data, and the impact damage to the middle slot of the scraper conveyor in a coal mine can be more severe during operation.

In addition, due to the complexity of the working environments in mines, especially with the passage of hydraulic support, the laying of a scraper conveyor may not be carried out according to the ideal design. However, to effectively reduce the impact damage to the middle trough and prolong its service life, the laying parameters of the scraper conveyor should be as close as to the optimal working conditions. In addition, there are still some other notable factors that can cause impact damage to the scraper conveyor. To further improve the performance of the scraper conveyor and to deepen related theoretical research, practice monitoring and online data collection remain to be conducted in the future.

## Supporting information

S1 File (XLS)

## References

[1] Zhang Y, Feng G, Zhang M, et al. Residual coal exploitation and its impact on sustainable development of the coal industry in China. Energy Policy 2016;96:534–541.

[2] Mao J, Yang XW, Cheng HY, Song QS. Analysis of dynamic characteristics of scraper conveyor. Journal of Machine Design 2018;35(6):47–53. (in Chinese with an English abstract)

[3] Shi ZY, Zhu ZC. Case study: Wear analysis of the middle plate of a heavy-load scraper conveyor chute under a range of operating conditions. Wear 2017;380/381:36–41.

[4] Xia R, Li B, Wang X, Yang ZJ, Liu LP. Screening the main factors affecting the wear of the scraper conveyor chute using the Plackett Burman method. Mathematical Problems in Engineering 2019; 2019(1):1–11.

[5] Grincova A, Andrejiova M, Mrasova D, Khouri S. Measurement and determination of the absorbed impact energy for conveyor belts of various structures under impact loading. Measurement 2019;131:362–371.

[6] Ge S, Wang Q, Wang J. The impact wear-resistance enhancement mechanism of medium manganese steel and its applications in mining machines. Wear 2017;376–377(B):1097–1104.

[7] Chen ZK, Lu SC, Song XB, Zhang HF, Yang WS, Zhou H. Effects of bionic units on the fatigue wear of gray cast iron surface with different shapes and distributions. Optics & Laser Technoloy 2015; 66: 166–174.

[8] Wang HC, Zhao WH, Sun DS, Guo BB. Mohr‐Coulomb Yield Criterion in Rock Plastic Mechanics. Chinese Journal of Geophysics 2012;55(6):733–741.

[9] Sun YL, Li QM, Lowe T, McDonald SA, Withers PJ. Investigation of strain-rate effect on the compressive behaviour of closed-cell aluminium foam by 3D image-based modelling. Materials & design 2016; 89: 215–224.

[10] Wang Y, Li JX, Wang JX. Research on impact characteristics of middle trough of scraper conveyor. Industry and Mine Automation 2019;45(04):19–23. (in Chinese with an English abstract)

[11] Hu DG. Mechanical system analysis and experimental research of scraper conveyor. Disseration. China University of Mining and Technology, Beijing, 2015.

[12] Jafari A, Nezhad VS. Employing DEM to study the impact of different parameters on the screening efficiency and mesh wear. Powder Technology: An International Journal on the Science and Technology of Wet and Dry Particulate Systems 2016;297:126–143.

[13] Zhang J, Zhang XB, Liu QH, et al. Effects of Load on Dry Sliding Wear Behavior of Mg-Gd-Zn-Zr Alloys[J]. Journal of Materials Science & Technology 2017;(7):645–651.

[14] Sardar S, Karmakar SK, Das D. High stress abrasive wear characteristics of Al 7075 alloy and 7075/Al2O3 composite. Measurement 2018;127:42–62.

[15] Mangwandi C, Adams MJ, Cheong YS, et al. The coefficient of restitution of different representative types of granules. Chemical Engineering Science 2007;62(1):437–450.

[16] Sinha R, Mukhopadhyay AK. Influence of particle size and load on loss of material in manganese-steel material: an experimental investigation. Archives of Metallurgy and Materials 2018;63(1):359–364.

[17] Reid AW, McAree PR, Meehan PA, et al. Longwall shearer cutting force estimation. Journal of Dynamic Systems, Measurement, and Control 2014;136(3):81–89.

[18] Xia R. Wear characteristics and prediction of middle groove of scraper conveyor under multi factor coupling. Dissertation. Taiyuan University of Technology, 2019.

[19] Yang XB, Li JX. The wear resistance of plasma cladding layers on the substrate Hardox450 of middle trough. IOP Conference Series: Materials Science and Engineering 2018;397(1):012–138.

[20] Yao YP, Gao ZP. Research on Impact Wear of Coal Falling Area in Middle Groove of Scraper Conveyor. Coal Mine Machinery 2021;42(3):35–37. (in Chinese with an English abstract)

[21] Gao ZP. Study on impact damage characteristics of coal falling area in the middle slot of scraper conveyor. Dissertation. Taiyuan University of Science & Technology, 2021. doi: 10.3390/polym12030648 32178318 PMC7182880

[22] Abdelwaly EA, Mohamed AA, El-Kosasy AM., Ayad MF. A comprehensive stability assessment of insulin degludec using new customized validated RP-HPLC and SEC-HPLC methods in an orthogonal testing protocol. Journal of Pharmaceutical and Biomedical Analysis 2021;203:114–175. doi: 10.1016/j.jpba.2021.114175 34098506

[23] Ghodki BM, Patel M, Namdeo R, Carpenter G. Calibration of discrete element model parameters: soybeans. Computational Particle Mechanics 2019;6:3–10.

[24] Oldal I, Safranyik F. Extension of silo discharge model based on discrete element metho. Journal of Mechanical Science and Technology 2015;29:3789–3796.

[25] Walker P, Kawalec W, Król R. Application of the discrete element method (DEM) for simulation of the ore flow inside the shaft ore bunker in the underground copper ire mine. Intelligent Systems in Production Engineering and Maintenance 2018;8(01):633–644.

[26] Osa JL, Sanchez JA, Ortega N, et al. Discrete-element modelling of the grinding contact length combining the wheel-body structure and the surface-topography models. International Journal of Machine Tools & Manufacture: Design, research & application 2016;110:43–54.

[27] Curry DR, Deng Y. Optimizing heavy equipment for handling bulk materials with Adams- EDEM co-simulation. International Conference on Discrete Element Methods 2016:1219–1224.

[28] Groger T, Katterfeld A. Application of the discrete element method in materials handling. Bulk Solids Handling: The International journal of Storing, Handling & Transporting Bulk 2007;27(1):17–22.

[29] China University of Mining and Technology. A wear detection device and method for middle groove of scraper conveyor: CN201710224906.5[P]. 2017-07-07.

[30] Yang HT. The experimental design of numerical analysis based on Matlab——Taking an example of singular value decomposition of matrix. Journal of Inner Mongolia University for Nationalities 2016; 31: 465–468.

